# COVID-19 outcome is not affected by anti-CD20 or high-titer convalescent plasma in immunosuppressed patients

**DOI:** 10.1038/s41598-023-48145-x

**Published:** 2023-12-01

**Authors:** Mary J. Kasten, Brian D. Lahr, Anusha Parisapogu, Zachary A. Yetmar, John C. O’Horo, Robert Orenstein, Pablo Moreno Franco, Raymund R. Razonable, Paschalis Vergidis, Aditya S. Shah, Mark J. Enzler, David J. Inwards, Philippe R. Bauer

**Affiliations:** 1 Division of Public Health, Infectious Diseases, and Occupational Medicine, Mayo Clinic, Rochester, MN 55905 USA; 2 Division of Clinical Trials and Biostatistics, Department of Quantitative Health Sciences, Mayo Clinic, Rochester, MN USA; 3 Infectious Diseases Research, Mayo Clinic, Rochester, MN USA; 4 Department of Infectious Disease, Integrated Hospital-Care Institute, Cleveland Clinic, Cleveland, OH USA; 5 Division of Pulmonary and Critical Care Medicine, Mayo Clinic, Rochester, MN 55905 USA; 6 Division of Infectious Diseases, Mayo Clinic, Phoenix, AZ USA; 7 Department of Critical Care Medicine, Mayo Clinic, Jacksonville, FL USA; 8 Division of Hematology, Emeritus Staff Center, Mayo Clinic, Rochester, MN USA

**Keywords:** Diseases, Health care, Medical research, Oncology

## Abstract

The role of severe acute respiratory syndrome coronavirus 2 (SARS-CoV-2) convalescent plasma in the treatment of Coronavirus Disease 2019 (COVID-19) in immunosuppressed individuals remains controversial. We describe the course of COVID-19 in patients who had received anti-CD20 therapy within the 3 years prior to infection. We compared outcomes between those treated with and those not treated with high titer SARS-CoV2 convalescent plasma. We identified 144 adults treated at Mayo clinic sites who had received anti-CD20 therapies within a median of 5.9 months prior to the COVID-19 index date. About one-third (34.7%) were hospitalized within 14 days and nearly half (47.9%) within 90 days. COVID-19 directed therapy included anti-spike monoclonal antibodies (n = 30, 20.8%), and, among those hospitalized within 14 days (n = 50), remdesivir (n = 45, 90.0%), glucocorticoids (n = 36, 72.0%) and convalescent plasma (n = 24, 48.0%). The duration from receipt of last dose of anti-CD20 therapy did not correlate with outcomes. The overall 90-day mortality rate was 14.7%. Administration of convalescent plasma within 14 days of the COVID-19 diagnosis was not significantly associated with any study outcome. Further study of COVID-19 in CD20-depleted individuals is needed focusing on the early administration of new and potentially combination antiviral agents, associated or not with vaccine-boosted convalescent plasma.

## Introduction

### Background and rationale

Coronavirus Disease 2019 (COVID-19) from severe acute respiratory syndrome coronavirus 2 (SARS-CoV-2) infection remains a serious threat to immune suppressed persons despite the availability of vaccination, improved understanding of its pathophysiology and management. Antiviral agents such as oral nirmatrelvir plus ritonavir^1^ and intravenous remdesivir attenuate the severity of COVID-19^2^ and immunomodulator agents including glucocorticoids^3–5^, Interleukine-6 inhibitors^6^, and Janus kinase inhibitors^7^ modulate the inflammatory response with reduction in mortality of severely ill patients. Anti-spike monoclonal antibodies have also been associated with a reduction in hospitalization in high-risk patients with early mild or moderate disease in the outpatient setting but their efficacy disappeared with the emergence of resistant SARS-CoV-2 variants^8–10^. The role of SARS-CoV2 convalescent plasma remains controversial^11^ but could be beneficial if given early and contains high titers of neutralizing antibodies^12^. On August 23, 2020, the U.S. Food and Drug Administration (FDA) issued an emergency use authorization (EUA) for investigational convalescent plasma for the treatment of COVID-19 in hospitalized patients^13^. On February 4, 2021, the FDA issued a revision of the EUA for COVID-19 convalescent plasma to limit the authorization to the use of high titer COVID-19 convalescent plasma only for the treatment of hospitalized patients with COVID-19 early in the disease course who have impaired humoral immunity and cannot produce an adequate antibody response^14^. The National Institutes of Health (NIH) guidelines for COVID-19 state that there is insufficient evidence to recommend either for or against the use of high-titer convalescent plasma for the treatment of COVID-19 in hospitalized or nonhospitalized patients who are immunocompromised^15^. Vaccination is now the mainstay of the response against the pandemic and has been authorized in the US since December 14, 2020, but as of March 1st, 2023, only 69.3% of the US population had completed their primary vaccination and only 16.2% have received an updated (bivalent) booster^16^.

Patients treated with anti-CD20 therapy (for a variety of diseases such as vasculitis or hematologic malignancies) may be unable to mount an antibody response to natural infection and vaccination. Accordingly, the efficacy of vaccines is attenuated in patients with hematologic conditions^17,18^. The most common drugs targeting the CD20 antigen are rituximab, obinutuzumab and ocrelizumab. A positive SARS-CoV-2 serology is associated with a faster resolution of viral shedding than with a negative serology^19^. Patients with negative SARS-CoV-2 serologies who have received anti-CD20 therapy are at risk of prolonged viral shedding^20^, smoldering infection, more severe clinical disease^21^, development of variants^22^, reactivation^23^ and protracted COVID-19 course^24^. They may be unable to mount a robust anti-inflammatory response due to B-cell depletion^25^. Currently, it is unclear if the prolonged viral shedding seen with anti-CD20 therapy leads to worse outcomes. For instance, in patients with vasculitis, the pre-existing use of glucocorticoids, but not rituximab, and the presence of comorbid chronic respiratory disease are the main factors found to be associated with poor outcome in one study^26^. Hematologic malignancy patients with COVID-19 have a higher mortality than the general population infected with COVID-19 (40% vs. 3.6%)^27–29^. These patients have been reported to improve with convalescent plasma^30–37^.

### Objectives

1. To describe the natural course of COVID-19 in patients previously treated with anti-CD20 therapy for diseases including vasculitis and hematologic malignancies. Our hypothesis was that patients who acquire COVID-19 preceded by recent anti-CD20 therapy can develop a prolonged course of COVID-19 which is not improved by remdesivir and immunomodulator agents alone.
2. To quantify the risk for each outcome (change in the World Health Organization (WHO) ordinal outcome scale, survival at 30 and 90 days, hospital-free days alive, and number of hospitalizations related to COVID-19) among patients with COVID-19 according to the time they had received anti-CD20 therapy. Our hypothesis was that patients who acquire COVID-19 may develop more severe illness and be less responsive to COVID-19 directed therapy when anti-CD20 therapy was given less than 6 months prior to contracting COVID-19.
3. To compare the outcome of patients with COVID-19 who have received anti-CD20 therapy and are treated with high titer convalescent plasma aiming at reaching passive seroconversion to those not treated with convalescent plasma. Our hypothesis was that patients treated with anti-CD20 therapy within the previous 6 months have a reduced chance of achieving a full recovery without passive SARS-CoV2 seroconversion from convalescent plasma therapy.

## Methods

This study followed the guidelines for reporting observational studies^38^.

### Study design

This was a retrospective cohort study of adult patients with newly diagnosed COVID-19 who received anti-CD20 therapy within 3 years prior to the development of COVID-19 illness at the initial phase of the pandemic. The aims were to describe the clinical course, management, and outcomes in patients treated with anti-CD20 therapy, correlate the time since last anti-CD20 treatment with outcomes; and, in the subgroup of patients hospitalized early for COVID-19, explore the potential benefits of convalescent plasma transfusion. “Time zero” was considered the index date when the patient first tested positive for COVID-19, and the time-dependent nature of convalescent plasma infusion was explicitly used in the outcome analyses. The primary outcome of interest was the change in WHO ordinal outcome score measured at 30-day and 90-day follow-ups; secondary outcomes included 90-day mortality and hospital-free days alive^39^.

### Setting

This study was performed at Mayo Clinic sites in Minnesota, Wisconsin, Florida, and Arizona. The period of recruitment spanned from September 01, 2020, to February 28, 2021. Exposure included anti-spike monoclonal antibodies, antivirals, immunomodulators and convalescent plasma whenever administered. The follow up was 90-days after the first positive SARS-CoV-2 PCR result.

### Participants

This study included all adults, 18-year-old and older, diagnosed for the first time with COVID-19 with a positive SARS-CoV-2 PCR, enrolled in the Mayo Clinic COVID-19 registry^40^, and previously treated for either vasculitis or hematologic malignancies with anti-CD20 therapy including rituximab, obinutuzumab, or ocrelizumab within the past 3 years.

A subgroup of patients who were hospitalized within 14 days of diagnosis was used in a secondary analysis for treatment comparison. These patients were categorized into two groups according to whether they received high titer convalescent plasma within 14 days from the index date. High titer convalescent COVID-19 plasma is based on serologic correlates of neutralizing activity and only plasma that met the FDA’s definition for high titer plasma was used^41^. The rationale was that hospitalized patients with COVID-19 who recently received anti-CD20 therapy are likely to have reached the inflammatory phase of COVID-19, while still having active viral replication, persistent shedding, mutations and lack or reduced antibody response. The administration of glucocorticoids alone may also prolong infection and prevent recovery^42^. Providing patients who are unable to mount an antibody response to SARS-CoV-2 with passive immunity using high titer convalescent plasma may lead to clinical and laboratory improvement^30^. Additionally, convalescent plasma has consistently been shown to reduce viral load^20,43^. We excluded patients who had declined to have their chart reviewed for research purposes.

The need for ethical approval was waived by Mayo Clinic Institutional Review Board (IRB # 21-001374) and registered with the U.S. National Library of Medicine: COVID-19 Infection in Patients Receiving Anti-CD20 Therapy; NCT04884477: https://clinicaltrials.gov/ct2/show/NCT04884477.

### Outcomes

The primary outcome of interest was the modified WHO ordinal outcome score^44^, a 7-level scale which we measured at baseline, and at 30-day and 90-day follow-ups. The primary end point for this study was the ordinal assessment at day 30. Secondary outcomes included the 90-day ordinal outcome assessment, as well as mortality and hospital-free days alive at 90-day follow up. Hospitalizations were limited to those related to COVID-19 infection.

### Data sources/measurement

Subjects were identified using an institutional registry of COVID-19 patients. Data collection was primarily based on manual review of the electronic medical records by several investigators. Disagreements were resolved by consensus.

### Study size

No a priori sample size calculation was performed.

### Statistical methods

Statistical analysis was performed using R version 4.0.3 (R Foundation for Statistical Computing, Vienna, Austria)^45^. Baseline characteristics are described with medians and interquartile ranges (IQR) for continuous variables and percentages for categorical variables. The index date a patient first tested positive for COVID-19 was considered “time zero” for all follow-up assessments. Frequency of intervening events (e.g., hospitalization, convalescent plasma transfusion) and mortality were estimated cumulatively over the 90-day follow-up period using the Kaplan–Meier estimator. The relationships between time since last anti-CD20 treatment and study outcomes (as measured by the WHO ordinal outcome scale, survival time, and hospital-free days alive) were expressed using Spearman’s rank correlation coefficient. Repeated assessments of the ordinal outcome scale for an individual were compared using the Wilcoxon signed-rank paired test. For all analyses, P < 0.05 was considered statistically significant.

Exploratory analysis was conducted on the subgroup of patients hospitalized in the first 14 days to investigate the patterns and potential benefits of convalescent plasma transfusion. For descriptive and practical purposes, we designated day 14 as a landmark time before which the exposure to treatment (convalescent plasma transfusion) was defined and after which the outcomes were measured. Before analyzing outcomes, a propensity analysis was undertaken to describe the patterns of treatment with convalescent plasma. The comparability of key baseline characteristics between treatment groups was assessed initially using standardized mean differences. The propensity score (i.e., the probability of receiving transfusion by day 14 given the patient’s baseline characteristics) was then estimated using multivariable logistic regression and later included as a covariate in the outcome models to adjust for confounding.

The risk of outcomes for hospitalized patients treated with versus without convalescent plasma by day 14 was assessed using semiparametric regression models. Specifically, we used the proportional odds ordinal logistic regression model to analyze the WHO ordinal outcome scale and hospital-free days alive after day 14, and the Cox proportional hazards regression model to analyze survival over 90 days. For modeling the ordinal outcome scale, an ordinal regression extension for repeated measurements was used to incorporate the 30-day and 90-day assessments into a single model. In particular, the model used a robust sandwich variance estimator to account for correlated responses from two observations on the same patient, with additional covariates included for the follow-up time and the baseline measure of the outcome. For modeling survival, we used an extended form of the Cox model for time-dependent covariates to assess the treatment effect over time, thereby allowing analysis of the entire 90-day survival curve. To be consistent with the landmark variable, our main time-dependent variable for convalescent plasma captured only transfusions given up to day 14; however, in a secondary Cox analysis, we considered a separate time-dependent variable for transfusion at any point during the 90-day follow-up period. To allow for nonlinear effects of covariates, the propensity score was modeled (on logit scale) with a linear tail-restricted cubic spline function, while the baseline ordinal outcome score was modeled as quadratic.

### Research involving human participants, data, or biological material

This was a retrospective study that was reviewed and deemed exempt by the Mayo clinic IRB, who waived the need for informed consent. All the methods were performed in accordance with relevant institutional guidelines and regulations. Participants who declined research authorization were not included.

## Results

**Figure 1:**
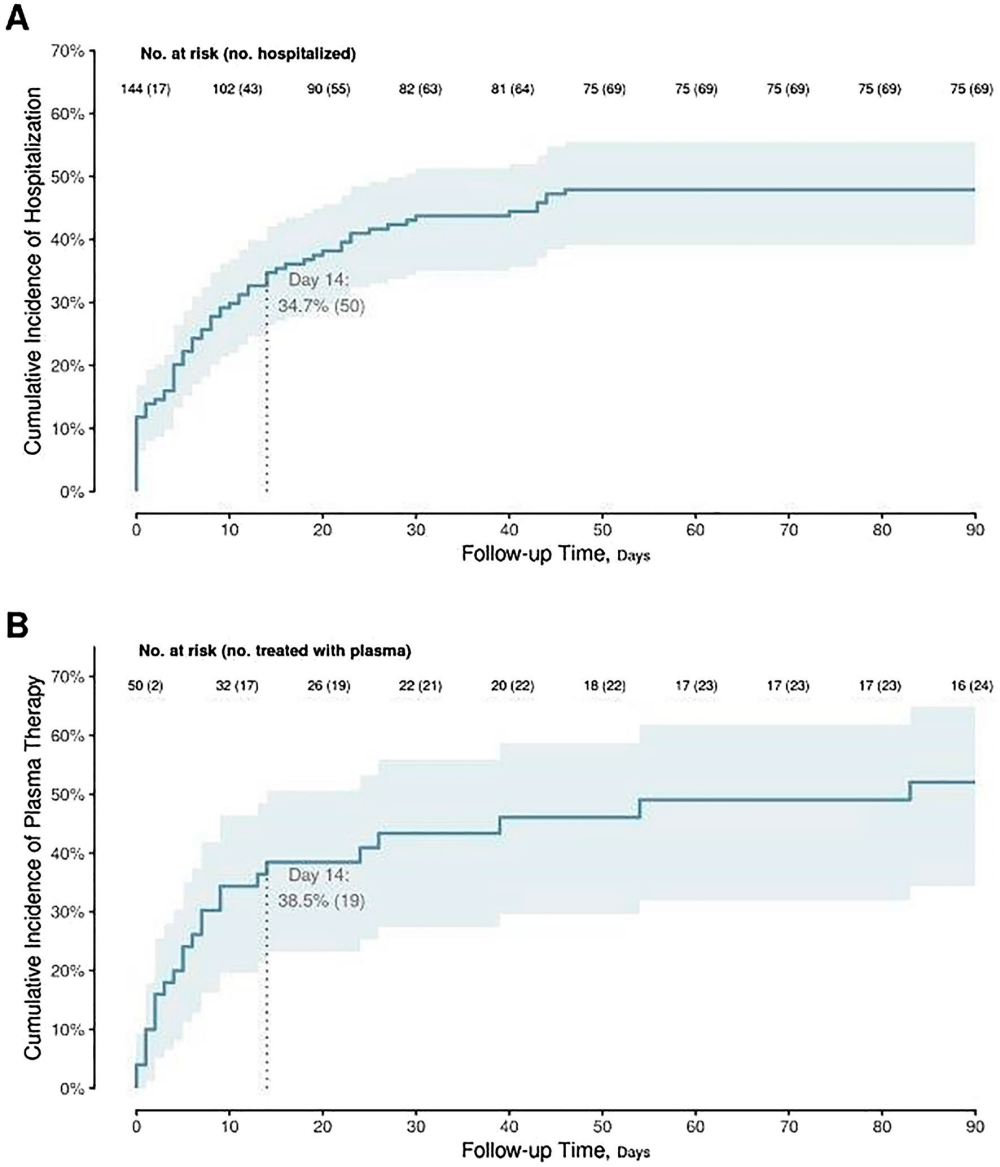
Cumulative rates of (**A**) hospitalization and (**B**) convalescent plasma (within hospitalized subgroup) across 90-day follow-up.

A total of 144 adults previously treated with anti-CD20 therapy contracted COVID-19 for the first time between September 1, 2020, and February 28, 2021 (Table 1). Median age was 63.6 years (IQR 54.0–74.5), 58.3% were male, 93.0% were White, and 97.1% were non-Hispanic. The last infusion of anti-CD20 therapy (rituximab, 92.4%, obinutuzumab, 7.6%) was a median of 5.9 months (2.1–15.7) prior to the date of laboratory-confirmed COVID-19. The most common indication for anti-CD20 treatment was hematologic malignancy (56.9%). Most of the patients were not vaccinated; only 3 patients received a single dose of COVID-19 vaccine, including one who contracted COVID-19 the same day. More than one-third of our cohort (n = 50, 34.7%) were hospitalized within 14 days of their positive test, and nearly half (n = 69, 47.9%) were hospitalized for COVID-19 at some point during the 90-day follow-up (Fig. 1). Thirty patients were treated as outpatient with one of two anti-spike monoclonal antibodies available at that time (20.8%), most of whom received bamlanivimab (80.0%) while the others received casirivimab-imdevimab (20.0%); seven of them had to be hospitalized and two of them subsequently received convalescent plasma (Fig. S1). Compared with baseline scores, a statistically significant worsening in the WHO ordinal outcome scale was noted at day 30 (P < 0.001) that persisted through day 90 (P = 0.006), with mean increases from baseline of 0.6 and 0.5 points, respectively for the whole cohort of 144 patients (Table 2; Fig. S2). The overall mortality rates at 30 and 90 days were 8.3% and 14.7%, respectively. No statistically significant correlations were noted between time since anti-CD20 treatment and the WHO ordinal outcome score at day 30 (Spearman correlation coefficient ρ = − 0.12, P = 0.141), hospital-free days alive (ρ = 0.12, P = 0.167) or overall days alive (ρ = 0.11, P = 0.176) during the 90-day follow-up.

**Table 1:** Baseline characteristics.

Characteristic	N	Overall (N = 144)
Age at diagnosis, years	144	63.6 (54.0–74.5)
Male sex	144	84 (58.3%)
Race	142	
White		132 (93.0%)
Black		4 (2.8%)
Asian		4 (2.8%)
Other		2 (1.4%)
Ethnicity: Hispanic	139	4 (2.9%)
COVID-19 vaccination single dose	144	3 (2.1%)
Body mass index, kg/m^2^	144	28.4 (24.1–32.6)
Indication for anti-CD20 treatment	144	
Hematologic malignancy		82 (56.9%)
Solid organ cancer		1 (0.7%)
Bone marrow transplantation		5 (3.5%)
Solid organ transplant		3 (2.1%)
Connective tissue disease		19 (13.2%)
Vasculitis		14 (9.7%)
Other		20 (13.9%)
Anti-CD20 drugs	144	
Rituximab		133 (92.4%)
Obinutuzumab		11 (7.6%)
Time since last anti-CD20 treatment, months	144	5.9 (2.1–15.7)
Co-morbidity	144	
Malignancy		98 (68.1%)
Renal disease		30 (20.8%)
Rheumatologic disease		29 (20.1%)
Chronic obstructive lung disease		29 (20.1%)
Diabetes mellitus		26 (18.1%)
Charlson index score	144	2 (2–4)
COVID-19 outcome scale, day 0	144	
1: Not hospitalized, no limitation in activity		110 (76.4%)
2: Not hospitalized, limitation in activity		17 (11.8%)
3: Hospitalized without oxygen		7 (4.9%)
4: Hospitalized with oxygen		4 (2.8%)
5: Noninvasive ventilation or high flow oxygen		6 (4.2%)
6: Invasive mechanical ventilation or ECMO		0 (0.0%)
7: Death		0 (0.0%)
Mean (median, IQR)		1.5 (1, 1–1)

Values represent median (quartile 1 to quartile 3) for continuous variables and frequency (percentage) for discrete variables, except when noted otherwise. N is the number of non-missing values.

**Table 2:** Clinical outcomes.

Outcome	N	Overall (N = 144)	Hospitalized (N = 50)
COVID-19 outcome scale, day 30	143		
1: Not hospitalized, no limitation in activity		83 (58.0%)	14 (28.6%)
2: Not hospitalized, limitation in activity		31 (21.7%)	12 (24.5%)
3: Hospitalized without oxygen		6 (4.2%)	1 (2.0%)
4: Hospitalized with oxygen		4 (2.8%)	3 (6.1%)
5: NIV or HFNO		6 (4.2%)	6 (12.2%)
6: IMV or ECMO		1 (0.7%)	1 (2.0%)
7: Death		12 (8.4%)	12 (24.5%)
Mean (median, IQR)		2.1 (1, 1–2)^a^	3.5 (2, 1–6)^c^
COVID-19 outcome scale, day 90	143		
1: Not hospitalized, no limitation in activity		115 (80.4%)	26 (53.1%)
2: Not hospitalized, limitation in activity		5 (3.5%)	5 (10.2%)
3: Hospitalized without oxygen		2 (1.4%)	0 (0.0%)
4: Hospitalized with oxygen		0 (0.0%)	0 (0.0%)
5: NIV or HFNO		0 (0.0%)	0 (0.0%)
6: IMV or ECMO		0 (0.0%)	0 (0.0%)
7: Death		21 (14.7%)	18 (36.7%)
Mean (median, IQR)		1.9 (1, 1–1)^a,b^	3.3 (1, 1–7)^c,d^
Cumulative mortality	144		
Days = 14		4 (2.8%)	4 (8.0%)
Days = 30		12 (8.3%)	12 (24.0%)
Days = 90		21 (14.6%)	18 (36.0%)
Hospital readmission, post-day 14	49	–	20 (40.8%)
Hospital-free days alive	143	90.0 (78.0–90.0)	75.0 (12.0–83.0)

Values represent median (quartile 1 to quartile 3) for continuous outcome variables and frequency (percentage) for binary/ordinal outcome variables, except when noted otherwise. N is the number of non-missing values. NIV: noninvasive ventilation; HFNO: high flow nasal oxygenation; IMV: invasive mechanical ventilation; ECMO: extracorporeal membrane oxygenation.

^a^P < 0.001 and P = 0.006, *vs.* baseline for day 30 and 90, respectively.

^b^P = 0.057, *vs.* day 30.

^c^P = 0.003 and P = 0.011, *vs.* baseline for day 30 and 90, respectively.

^d^P = 0.362, *vs.* day 30.

In the subset analysis of 50 patients hospitalized by day 14 (Table S1), laboratory measurements within the first 3 days of admission indicated lymphopenia (absolute lymphocytes, 0.7 [0.5–1.1] × 10^9^/L), with inflammation present but relatively mild as indicated by levels of C-reactive protein (62.2 [33.6–99.1] mg/L), d-dimer (900 [531–1459] ng/mL FEU) and ferritin (559 [250–890] mcg/L). Among hospitalized patients, COVID-19-directed therapy included remdesivir (90.0%), glucocorticoids (72.0%) and the percentage of patients receiving convalescent plasma, by 14 and 90 days, was 38.0% and 48.0%, respectively. During the 90-day follow-up, rehospitalization for COVID-19 was frequent (40.8%).

**Figure 2:**
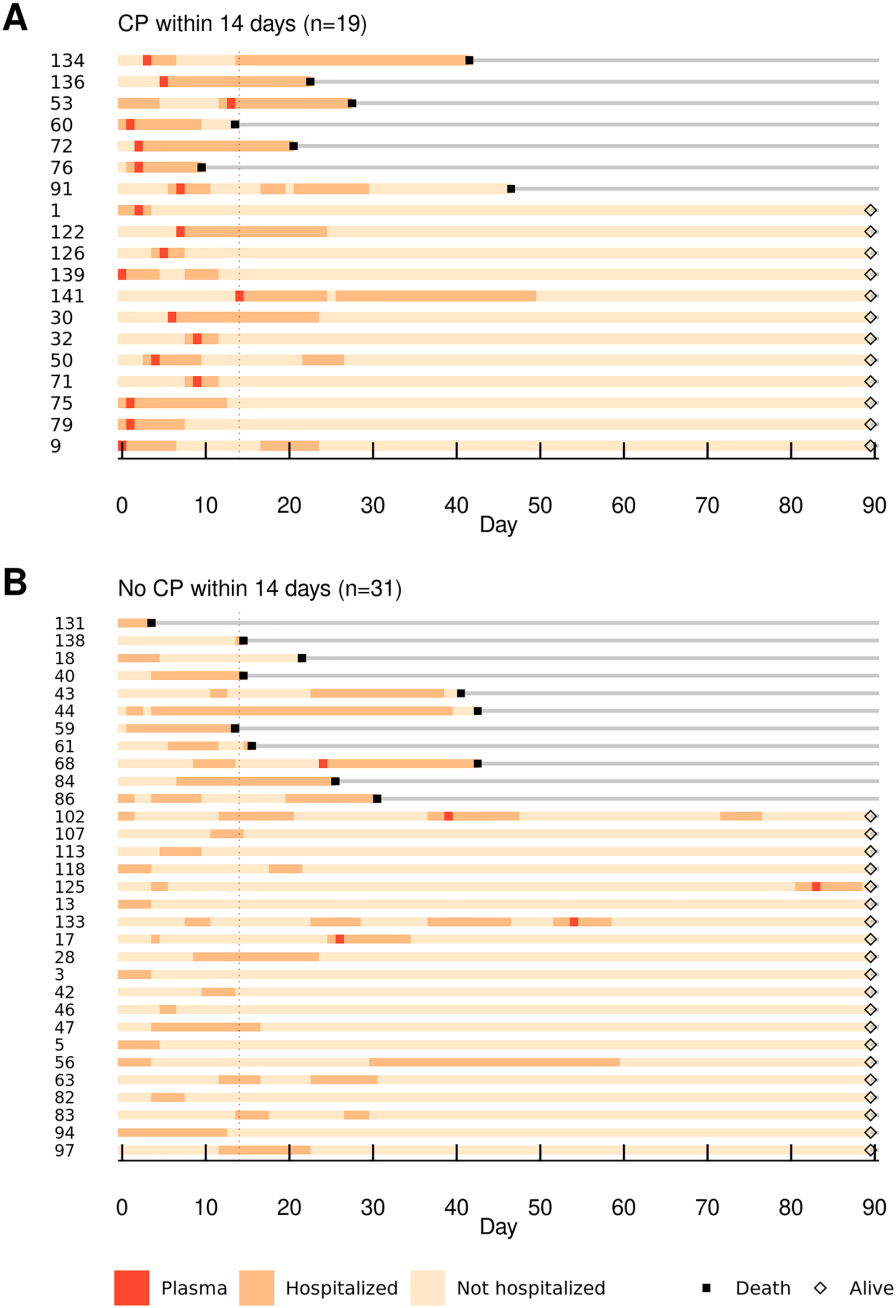
Hospital event charts for patients treated with (**A**) and without (**B**) convalescent plasma (CP) in the first 14 days. Each horizontal line in the chart represents an individual patient, while the vertical red line is used to reference day 14. Numbers listed on the left are study-assigned subject numbers. Note that subject number 79 was lost to clinical follow-up following hospital discharge on day 7, although the patient was known to be alive on day 90.

Nineteen hospitalized patients were transfused with convalescent plasma in the first 14 days of follow-up and 31 hospitalized patients were not (Fig. 2). Comparison of baseline descriptors in these 2 groups revealed some imbalances, with a tendency for those receiving convalescent plasma by day 14 to be younger, female, and having shorter time to hospitalization from diagnosis of COVID-19 and more recent anti-CD20 treatment (standardized difference > 0.25 each) (Table S2). Outcomes are reported in these 2 groups (Table 3), (Fig. S3). Propensity-adjusted regression analyses did not demonstrate any significant effects of convalescent plasma on the primary or secondary outcomes (Table S3).

**Table 3:** Clinical outcomes according to 14-day treatment groups with or without convalescent plasma (CP).

Outcome	N	CP (N = 19)	No CP (N = 31)
COVID outcome scale, day 30	49*		
1: Not hospitalized, no limitation in activity		5 (27.8%)	9 (29.0%)
2: Not hospitalized, limitation in activity		5 (27.8%)	7 (22.6%)
3: Hospitalized without oxygen		0 (0.0%)	1 (3.2%)
4: Hospitalized with oxygen		1 (5.6%)	2 (6.5%)
5: Noninvasive ventilation or high flow oxygen		1 (5.6%)	5 (16.1%)
6: Invasive mechanical ventilation or ECMO		1 (5.6%)	0 (0.0%)
7: Death		5 (27.8%)	7 (22.6%)
Mean (median, IQR)		3.6 (2, 1–7)	3.5 (2, 1–5)
COVID outcome scale, day 90	49*		
1: Not hospitalized, no limitation in activity		8 (44.4%)	18 (58.1%)
2: Not hospitalized, limitation in activity		3 (16.7%)	2 (6.5%)
3: Hospitalized without oxygen		0 (0.0%)	0 (0.0%)
4: Hospitalized with oxygen		0 (0.0%)	0 (0.0%)
5: Noninvasive ventilation or high flow oxygen		0 (0.0%)	0 (0.0%)
6: Invasive mechanical ventilation or ECMO		0 (0.0%)	0 (0.0%)
7: Death		7 (38.9%)	11 (35.5%)
Mean (median, IQR)		3.5 (2, 1–7)	3.2 (1, 1–7)
Cumulative mortality	50		
Days = 14		2 (10.5%)	2 (6.5%)
Days = 30		5 (26.3%)	7 (22.6%)
Days = 90		7 (36.8%)	11 (35.5%)
Post-14-day hospital outcomes			
Hospital readmission	45	7 (43.8%)	13 (44.8%)
Hospital-free days alive	45	67.5 (15.8–76.0)	66.0 (10.0–76.0)

Values represent median (quartile 1 to quartile 3) for continuous outcome variables and frequency (percentage) for binary/ordinal outcome variables. N is the number of non-missing values.

*One patient was lost to follow up early on and was therefore not included in the responses for the COVID outcome scale.

## Discussion

### Key results

During the initial phase of the pandemic, 144 mostly unvaccinated patients, who were previously treated with anti-CD20 therapy, contracted de novo COVID-19. A fifth of patients received anti-spike monoclonal antibodies. Half of the patients were hospitalized within 90 days; of those hospitalized, most received remdesivir and glucocorticoids, and about half of them ultimately received convalescent plasma. At day 90, the overall mortality was low, and most patients were clinically doing well, not hospitalized, and off oxygen. The use of convalescent plasma in the first 14 days was not associated with a better outcome.

### Interpretation

This study underscores the unique challenges faced by clinicians during the early stages of the pandemic in treating CD20-depleted immunocompromised patients, many of whom had received anti-CD20 treatment within six months prior to contracting COVID-19. These patients generally have an undetectable CD20 level and are at high risk for adverse outcomes from COVID-19, even if vaccinated^46^. We chose to study this highly humorally immunosuppressed group with COVID-19 before widespread availability of SARS-CoV-2 vaccines as we hypothesized that they may be the group to benefit most from convalescent plasma which we were unable to demonstrate. Many of these patients had an underlying hematologic malignancy which may confer additional immunosuppression independent of B cell depletion. This may help explain why there was no correlation with the last dose of anti-CD20 therapy on outcomes. The lack of correlation between COVID-19 severity and timing of rituximab prior to infection has also been found in other studies^47–49^. The high proportion of patients treated with remdesivir is consistent with the known benefit of available antiviral agents, especially when given early to high-risk patients. A recent study found that high titer convalescent plasma reduced mortality in patients with COVID-19 ARDS when given within 5 days of the initiation of invasive mechanical ventilation^50^, but, the proportion of patients treated with remdesivir was very low (< 6%). Our findings suggest that patients hospitalized for COVID-19 infection in this immunosuppressed cohort may exhibit persistent viral shedding and comparatively low inflammation, as indicated by relatively low inflammation markers within three days of initial hospitalization. This may justify a prolonged course of antiviral agents as currently suggested by the NIH^15^. The high rate of rehospitalization for COVID-19 suggests a subacute and indolent infection pattern in this population, which raises concerns about the potential emergence of variants of concern due to ongoing or smoldering infection.

### Limitations

This study has several limitations. This is a retrospective observational study that is subject to the inherent limitations and biases of such designs. Analysis exploring the therapeutic benefit of convalescent plasma was performed only among a limited subset of the study population. Given this small sample and the low observed mortality rates, our study was underpowered to detect treatment differences in survival. Regarding the indication for prior anti-CD20 therapy (hematological versus non hematological), the hospitalized patients who received plasma within 14 days of COVID diagnosis and those who did not were reasonably balanced. However, we did note some imbalances in the time since last anti-CD20 treatment, Charlson index score, and in some of the individual comorbid conditions in the Charlson score including chronic obstructive lung disease, and diabetes (Table S2). However, the final outcomes analyses account for the differences in time since last anti-CD20 treatment and Charlson Index (as well as in age, sex, time to hospital admission) by use of propensity score adjustment with inclusion of the propensity score as a covariate in these regression models. Even though the level of anti-spike or receptor-binding domain (RBD) titers were not provided, only convalescent plasma with high titers could be delivered by the transfusion center as per FDA mandate. Furthermore, plasma treatment was neither randomized nor defined at the time of diagnosis, and attempting to compare dynamic, non-random treatment regimens is difficult. Our analysis incorporated time-dependent plasma measures and propensity adjustment techniques, although the sample size limited the number of baseline factors included in the propensity score. The use of anti-spike monoclonal antibodies was limited, as their use was restricted to patients diagnosed early and able to initiate treatment within 7 days of onset of symptoms. Convalescent plasma was not authorized in the outpatient setting at that time and therefore was only administered to inpatients who may have also received remdesivir, which is now proven effective when given early^2^. The potential synergistic effect of convalescent plasma when combined with antiviral therapies requires further investigation. The hypothesis that administering convalescent plasma at an earlier stage in outpatient settings for severely immunosuppressed patients may be promising but warrants additional research^51^. Vaccine-boosted convalescent plasma may be useful when there is high resistance to anti-spike monoclonal antibodies or particularly if variants resistant to current antivirals emerge^52^. Although high titer convalescent plasma was used, the actual composition of antibodies is unpredictable, limiting conclusion regarding its effectiveness. Viral load and cycle threshold were not measured. CD20 count was not measured, and the actual degree of immunosuppression is not known. Semi-quantitative COVID-19 antibody testing for nucleocapsid protein was rarely performed in this study. Moreover, a positive test after convalescent plasma does not guarantee passive immunity. Finally, this study included patients before vaccines were widely available. Even in patients with impaired immunity, vaccination has proven to be beneficial^53^.

### Generalizability

This study was limited to a single large institution, but it included several medical centers across 4 states in the U.S. increasing its external validity. Also, the state of care has dramatically changed with new antivirals, vaccinations, and improved inpatient care.

## Conclusion

Patients receiving anti-CD20 therapy with COVID-19 infection frequently needed hospitalization and often developed ongoing or smoldering infection. With the incomplete adherence to vaccination in the general population, the resistance to previously authorized anti-spike monoclonal antibodies, and the uncertainty about the efficacy of convalescent plasma against future variants, further study of COVID-19 in CD20-depleted individuals is needed that focuses on the early administration of new and potentially combination antiviral agents, associated or not with vaccine-boosted convalescent plasma^54,55^.

### Supplementary Information

Supplementary Figure S1.

Supplementary Figure S2.

Supplementary Figure S3.

Supplementary Information 4.

## Data Availability

Data will be available upon reasonable request by writing to the corresponding author. We will ensure data is completely deidentified before it is shared.

## Abbreviations

COVID-19: Coronavirus disease 2019
EUA: Emergency use authorization
FDA: Food and Drug Administration
IQR: Interquartile ranges
NIH: National Institutes of Health
SARS-CoV-2: Severe acute respiratory syndrome coronavirus 2
WHO: World Health Organization
