# Supplementary figures and images for: COVID-19 outcome is not affected by anti-CD20 or high-titer convalescent plasma in immunosuppressed patients
Source: Sci Rep. 2023 Dec 1;13:21249. doi: 10.1038/s41598-023-48145-x (PMC10692159; doi:10.1038/s41598-023-48145-x)

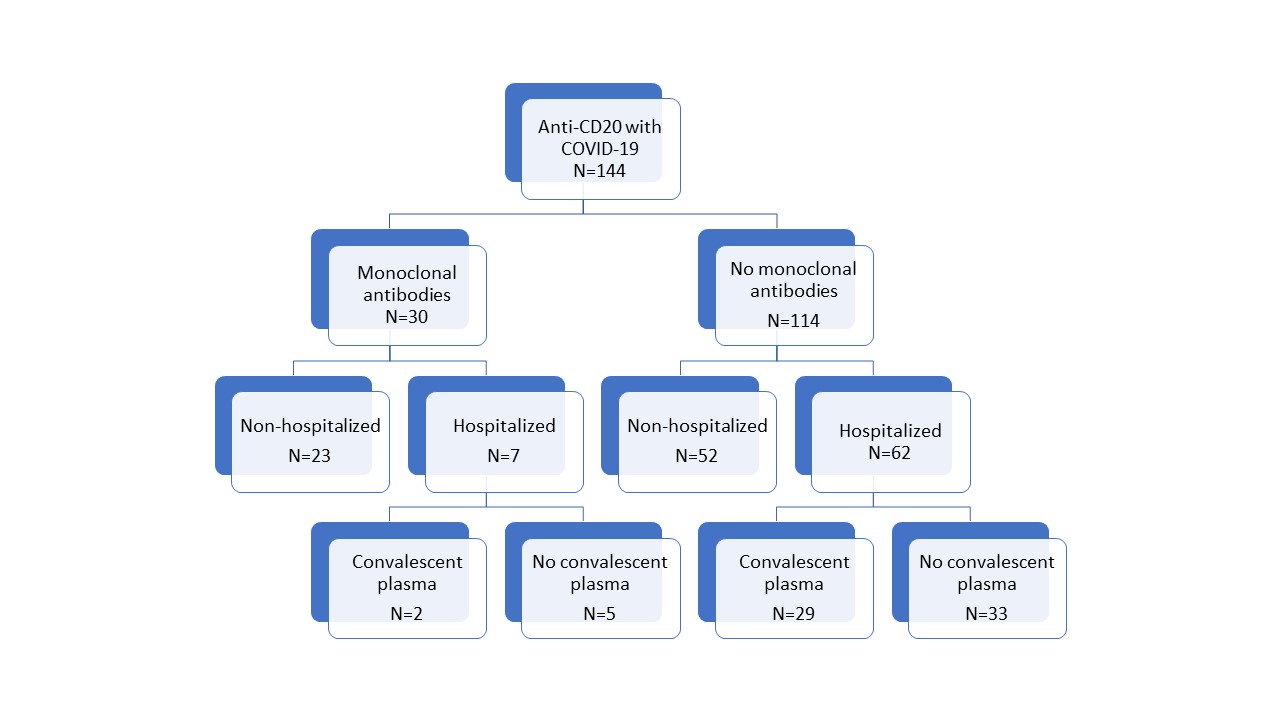

Supplement: Supplementary file 1 — Supplementary Figure S1. [file 41598_2023_48145_MOESM1_ESM.jpg]

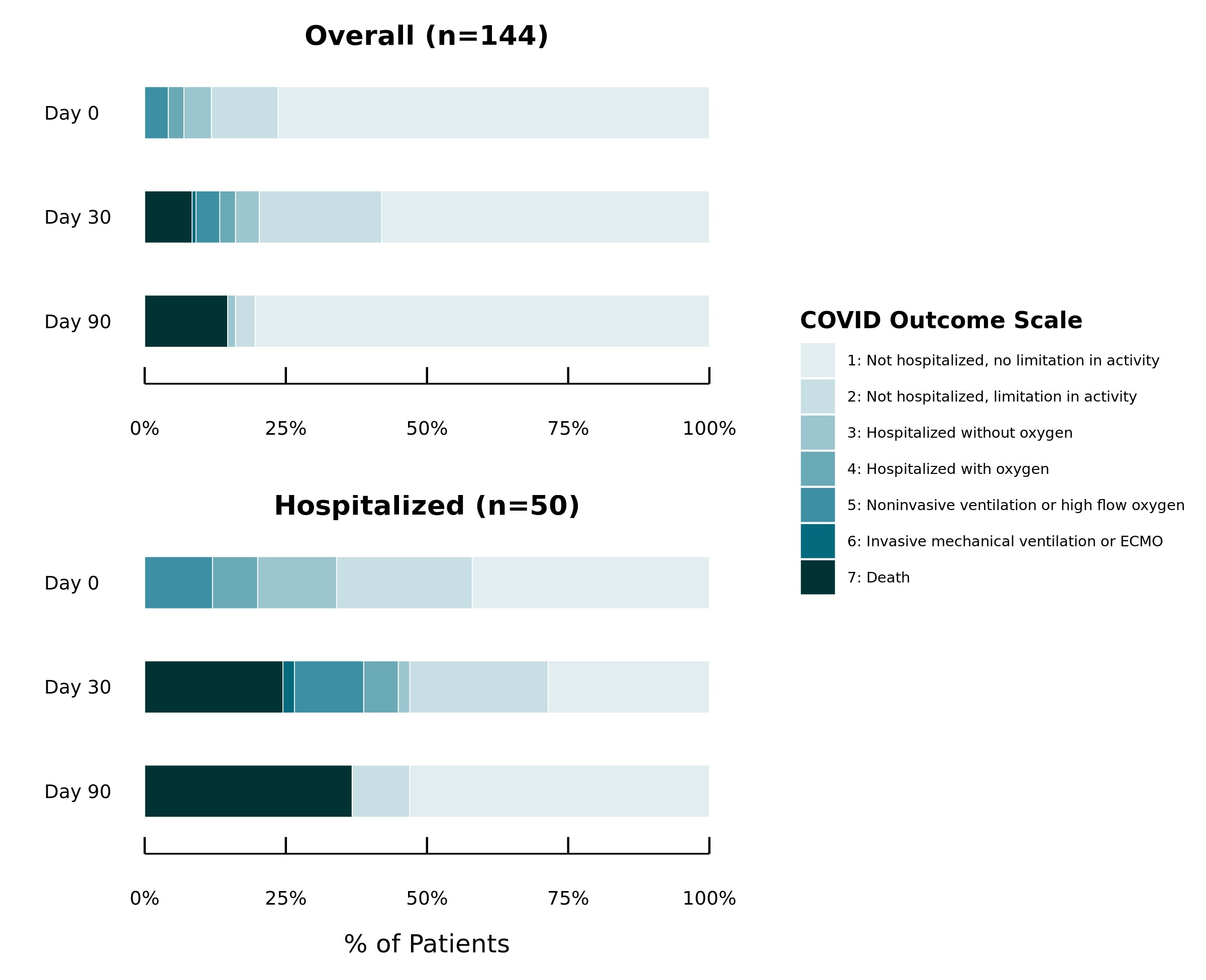

Supplement: Supplementary file 2 — Supplementary Figure S2. [file 41598_2023_48145_MOESM2_ESM.jpeg]

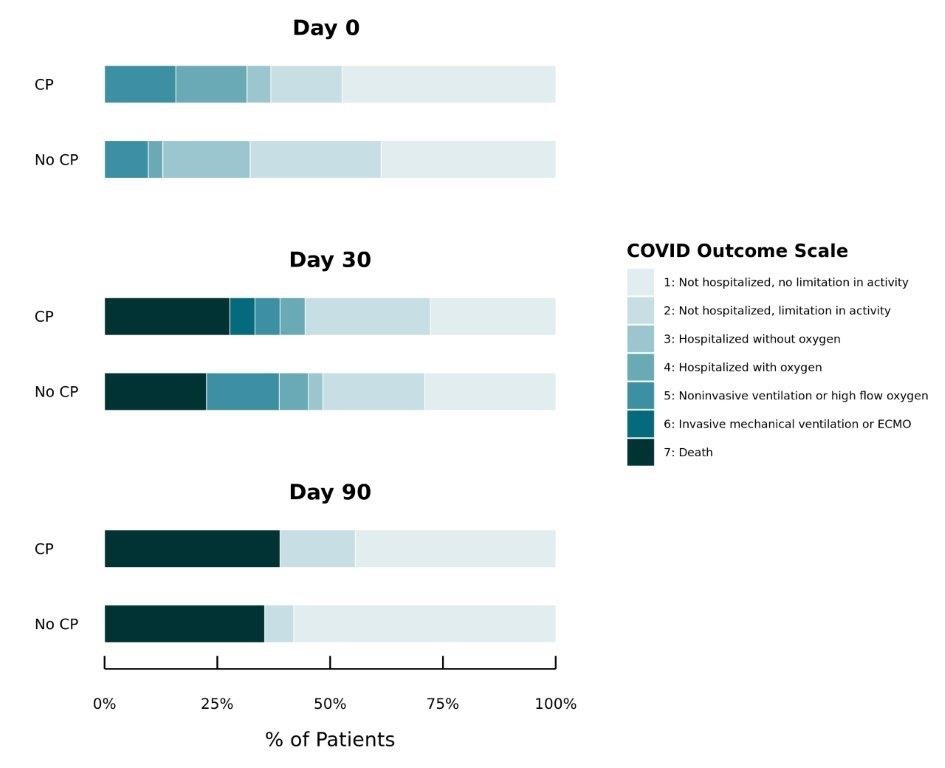

Supplement: Supplementary file 3 — Supplementary Figure S3. [file 41598_2023_48145_MOESM3_ESM.jpg]
