# Supplementary material for: COVID-19 outcome is not affected by anti-CD20 or high-titer convalescent plasma in immunosuppressed patients
Source: Sci Rep. 2023 Dec 1;13:21249. doi: 10.1038/s41598-023-48145-x (PMC10692159; doi:10.1038/s41598-023-48145-x)
Supplement: Supplementary file 4 — Supplementary Information 4. [file 41598_2023_48145_MOESM4_ESM.docx]

**Supplemental Materials**

**Table S1: Intervening exposures in subgroup (n=50) hospitalized within 14 days**

| **Characteristic** | **N** | **Hospitalized  (N=50)** |
| --- | --- | --- |
| No. of hospitalizations | 50 |  |
| 1 |  | 30 (60.0%) |
| 2 |  | 16 (32.0%) |
| 3 |  | 2 (4.0%) |
| 4 |  | 2 (4.0%) |
| Hospital readmission | 49 | 20 (40.8%) |
| Convalescent plasma within 14 days | 50 | 19 (38.0%) |
| Total no. of transfusions | 18 | 2.0 (1.0-2.8) |
| Convalescent plasma within 90 days | 50 | 24 (48.0%) |
| Total no. of transfusions | 23 | 2.0 (1.0-3.0) |
| Remdesivir within 90 days | 50 | 45 (90.0%) |
| Glucocorticoids within 90 days | 50 | 36 (72.0%) |
| *Laboratory measures (within 3 days of first admission)* |  |  |
| C-Reactive Protein, mg/L | 46 | 62.2 (33.6-99.1) |
| D-dimer, ng/mL FEU | 45 | 900 (531-1459) |
| Ferritin, mcg/L | 39 | 559 (250-890) |
| Hemoglobin, g/dL | 49 | 12.0 (10.8-13.4) |
| White Blood Count, x10^9/L | 49 | 5.8 (3.7-8.8) |
| Absolute neutrophils, x10^9/L | 49 | 3.8 (2.6-6.1) |
| Absolute lymphocytes, x10^9/L | 48 | 0.7 (0.5-1.1) |
| Absolute monocytes, x10^9/L | 48 | 0.6 (0.3-0.7) |
| Platelets, x10^9/L | 49 | 193 (117-227) |
| Creatinine, mg/dL | 49 | 1.0 (0.8-1.2) |
| EGFR, mL/min/BSA | 48 | 64.5 (56.8-84.2) |
| Aspartate aminotransferase, U/L | 48 | 39.5 (30.0-65.0) |
| Alanine transaminase, U/L | 48 | 32.0 (18.8-57.0) |
| Values represent median (quartile 1 to quartile 3) for continuous variables and frequency (percentage) for discrete variables. N is the number of non-missing values. FEU: fibrinogen equivalent units; EGFR: estimated glomerular filtration rate; BSA: body surface area | | |

**Table S2: Comparison of baseline descriptors by 14-day treatment with or without convalescent plasma (CP) therapy**

| **Characteristic** |  | **CP (N=19)** | **No CP (N=31)** | **SMD** |
| --- | --- | --- | --- | --- |
| Age at diagnosis, years |  | 61.0 (51.4-77.5) | 67.5 (59.2-79.3) | 0.272 |
| Male sex |  | 7 (36.8%) | 19 (61.3%) | 0.504 |
| Body mass index, kg/m² |  | 25.0 (23.4-31.7) | 30.0 (25.6-32.3) | 0.188 |
| Disease group: non-hematologic |  | 8 (42.1%) | 14 (45.2%) | 0.062 |
| Time since last anti-CD20 treatment, months |  | 4.4 (2.2-10.2) | 5.1 (2.1-18.8) | 0.444 |
| Malignancy |  | 13 (68.4%) | 19 (61.3%) | 0.150 |
| Renal disease |  | 5 (26.3%) | 9 (29.0%) | 0.061 |
| Rheumatoid disease |  | 4 (21.1%) | 6 (19.4%) | 0.042 |
| Chronic obstructive lung disease |  | 2 (10.5%) | 9 (29.0%) | 0.478 |
| Diabetes |  | 3 (15.8%) | 9 (29.0%) | 0.322 |
| Charlson index score |  | 2 (2-4) | 3 (2-6) | 0.310 |
| COVID-19 outcome scale, day 0^^^ |  | 2.4 (2, 1-4) | 2.2 (2, 1-3) | 0.143 |
| Time-to-14 days-hospital admission, days |  | 3 (0-6) | 4 (0-9) | 0.337 |
| Propensity score* |  | 0.47 (0.33-0.59) | 0.32 (0.22-0.44) | 0.735 |
| Values represent median (quartile 1 to quartile 3) for continuous variables and frequency (percentage) for discrete variables, except when noted otherwise. SMD is the absolute standardized mean difference.  ^ Values are reported as mean (median, quartile 1 to quartile 3)  * Propensity scores for CP therapy, based on the predicted probability of receiving CP therapy within 14 days of confirmed COVID-19, were derived from a logistic regression model that included age, sex, time since last anti-CD treatment, Charlson Index Score, and time to hospital admission. | | | | |

**Table S3: Propensity-adjusted effect of 14-day plasma on outcome**

|  | |  |  | **Propensity-Adjusted Effect of 14-Day Plasma** | |
| --- | --- | --- | --- | --- | --- |
| **Outcome** | **Number with available data** |  |  | **Hazard/Odds Ratio (95% Confidence Interval)** | **P-value** |
| 30-/90-day COVID outcome scale | 49 |  |  | 1.98 (0.65 - 6.03) | 0.232 |
| 90-day mortality *^a^* | 50 |  |  | 1.08 (0.38 - 3.04) | 0.883 |
| Hospital-free days, day 14 to 90 | 45*^b^* |  |  | 0.87 (0.28 - 2.71) | 0.805 |
| *^a^* Sensitivity analysis using a time-dependent indicator variable for receiving convalescent plasma at any point during the 90-day follow-up (including 5 patients transfused after day 14) showed a similar non-significant effect: HR 1.25, 95% CI 0.46-3.42, P=0.665.  *^b^* Analysis excluded 5 patients without >14-day follow-up (4 died, 1 was lost to follow-up of non-mortality outcomes) | | | | | |
|  | | | | | |

**Legends:**

**Figure S1: Flowchart**

**Figure S2: Clinical status at study days 0, 30, and 90, overall (n=144) and in the subgroup (n=50) hospitalized within 14 days**

**Figure S3: Differences in clinical status at days 0, 30, and 90 according to 14-day treatment with or without convalescent plasma (CP)**
